# Laparoscopic pyeloplasty for newborns with severe hydronephrosis

**DOI:** 10.3389/fped.2024.1343211

**Published:** 2024-03-14

**Authors:** Tong Shi, Weihua Lao, Keyu Ouyang, Yueqing Chen, Yikui Zhang, Jiayao Luo, Shuhan Chen

**Affiliations:** Pediatric Urology, Guangdong Women and Children Hospital, Guangzhou, China

**Keywords:** laparoscopy, neonatal period, ureteropelvic junction, hydronephrosis, surgery

## Abstract

**Aim:**

We aimed to investigate the short-term efficacy and safety of laparoscopic pyeloplasty for treating newborns with severe hydronephrosis due to ureteropelvic junction obstruction (UPJO).

**Methods:**

A retrospective analysis was performed on 16 newborn patients with severe neonatal hydronephrosis who underwent laparoscopic pyeloplasty at our hospital from January 2021 to November 2022. All patients were regularly followed up. Laparoscopic pyeloplasty with double J stent placement was performed after the presence of severe hydronephrosis was confirmed.

**Results:**

Among the 16 pediatric patients (13 males, 3 females), the left side was affected in 13 cases and the right side in 3. The average age at surgery was 9.50 (8.50–12.00) days, with an average weight of 3.30 ± 0.95 kg. Laparoscopic pyeloplasty was performed in all cases without the need for open conversion. The mean surgery duration was 292.06 ± 73.60 min, with minimal blood loss (2.50, 2.00–5.00 ml). Postoperative hospital stays averaged 13.44 ± 4.70 days. No anastomotic leakage occurred, and follow-ups at 1, 3, 6, and 12 months showed no stent displacement, except for one case where the stent was removed at 1 month, and the others at 3 months. There were no cases of worsened hydronephrosis, except for one with renal atrophy at the 6-month follow-up. Changes in renal pelvis anteroposterior diameter exhibited a time effect (*F* = 49.281, *P *< 0.001), with significant differences at 1, 3, 6, and 12 months postoperatively compared to preoperative values (*P *< 0.05). Notably, differences were observed between 6 and 3 months, as well as between 12 and 3 months postoperatively (*P *< 0.05). Similarly, renal parenchymal thickness changes showed a time effect Pediatric urology, Guangdong Women and Children Hospital, Guangzhou, China (*F* = 49.281, *P *< 0.001), with significant differences at 1, 3, 6, and 12 months postoperatively compared to preoperative values (*P *< 0.05). Significant differences were also noted between 6 and 1 month, as well as between 12 and 1 month postoperatively (*P *< 0.05). There was one case of urinary tract infection after surgery, and no case of recurrence was observed.

**Conclusion:**

Severe neonatal hydronephrosis must be treated promptly. Laparoscopic pyeloplasty is a safe and feasible treatment with minimal complications for newborn patients with severe hydronephrosis due to UPJO.

## 1 Introduction

Antenatal hydronephrosis (PNH) is a prevalent cause of renal and urinary tract abnormalities during the perinatal period, with an incidence of 1%–5% (1, 2). The diagnosis of PNH is made according to the results of prenatal and postnatal renal ultrasound (US). In the fetal US, PNH is defined as an anteroposterior diameter (APD) of the renal pelvis of ≥4 mm when the gestational age is less than 33 weeks and ≥7 mm when the gestational age is ≥33 weeks. Persistent postnatal urinary tract dilation is a crucial indicator, and an APD > 10 mm is considered abnormal (3).

The common cause of PNH is often physiological, whereas pathological hydronephrosis caused by ureteropelvic junction obstruction (UPJO) accounts for approximately 85%–90% of cases. Furthermore, many children with UPJO may need surgery during infancy to relieve the obstruction and prevent the deterioration of renal functions (4). Despite remarkable advancements in the diagnosis and treatment of several urinary tract system anomalies, substantial controversy exists regarding the treatment strategies for neonatal patients with PNH. Severe hydronephrosis is primarily treated via percutaneous nephrostomy or open surgery. However, laparoscopic surgery for the treatment of neonatal hydronephrosis is not well reported. Herein, we performed a single-center retrospective analysis to investigate the postoperative renal morphology following laparoscopic pyeloplasty in newborn patients with severe neonatal hydronephrosis. We have also reported the feasibility and clinical efficacy of laparoscopic treatment for severe neonatal hydronephrosis.

## 2 Clinical data

A retrospective analysis of clinical data was performed on newborns with severe hydronephrosis who were treated at our hospital from January 2021 to November 2022. The inclusion criteria were as follows: ① detected by prenatal US and confirmed by postnatal color Doppler US examination within 48 h of birth, with a diagnosis of UPJO, characterized by an APD of the renal pelvis ≥20 mm and classified as Society of Fetal Urology (SFU) grade 4 and ② age at the time of surgery <1 month. The exclusion criteria were as follows: the presence of other urinary tract anomalies, including bladder–ureter reflux, ureteral stenosis at other sites, duplex kidneys, severe hepatic or renal dysfunction, postoperative recurrence of UPJO, and cases where parents refused surgery or patients who did not comply to the follow-up period. Finally, we included 16 cases of severe PNH in newborns.

## 3 Surgical methods

**Figure 1:**
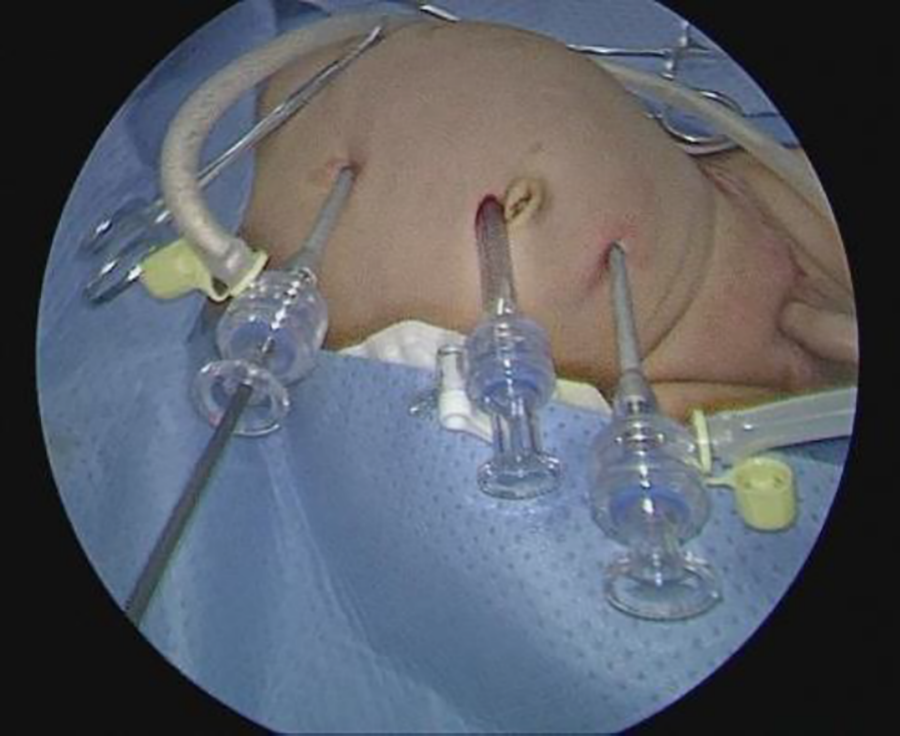
Position of the trocar.

**Figure 2:**
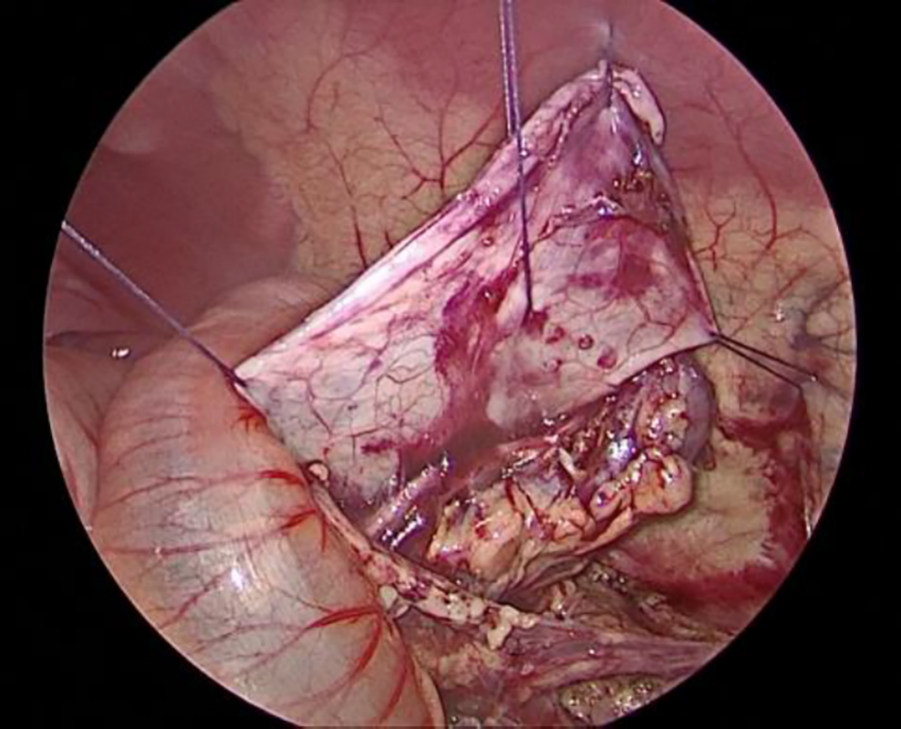
Elevation and suspension of the renal pelvis.

**Figure 3:**
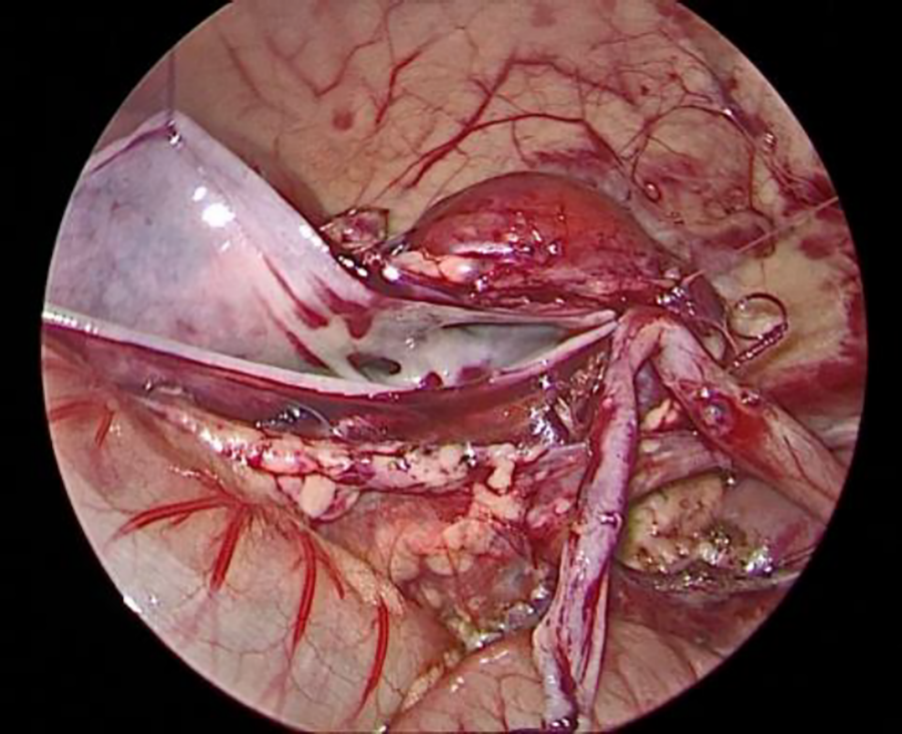
Suturing the lowest point of the renal pelvis to the ureter.

**Figure 4:**
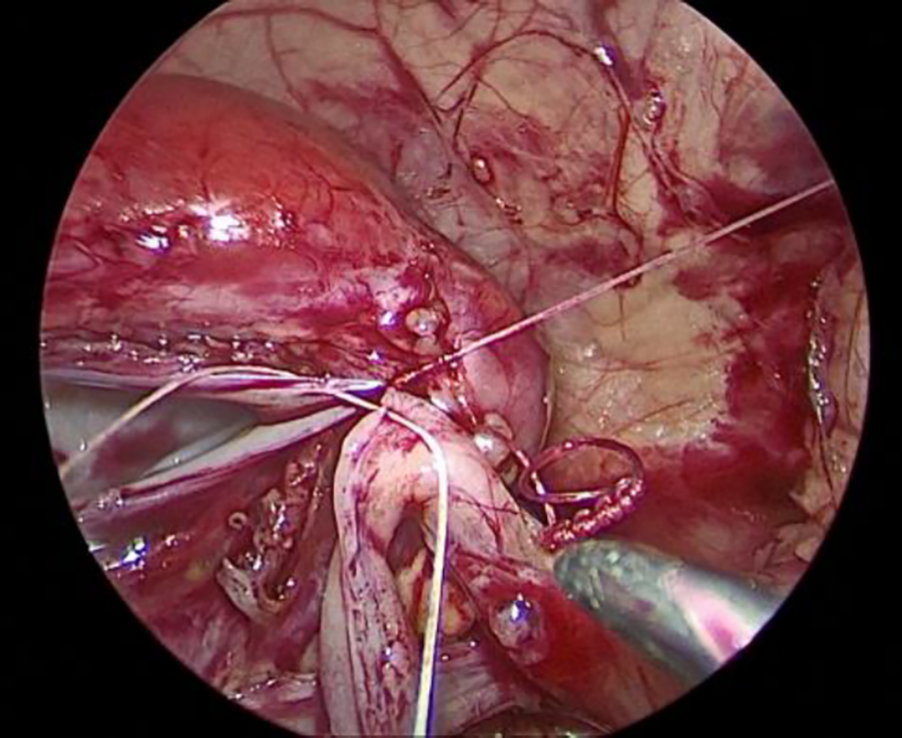
Width of the anastomosis between the ureter and the renal pelvis.

**Figure 5:**
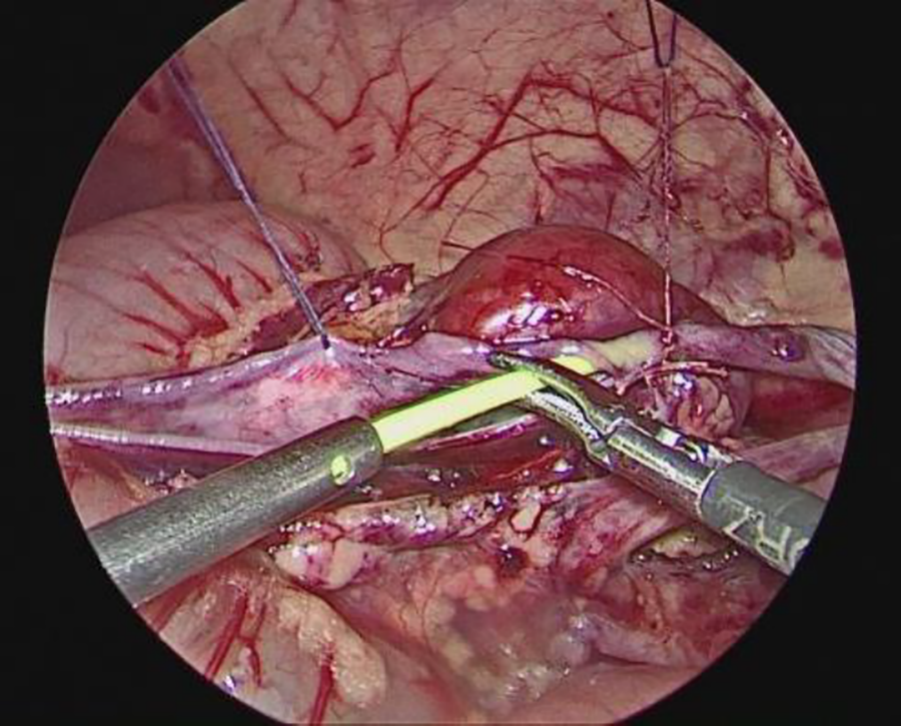
Placement of the double J stent under laparoscopy.

**Figure 6:**
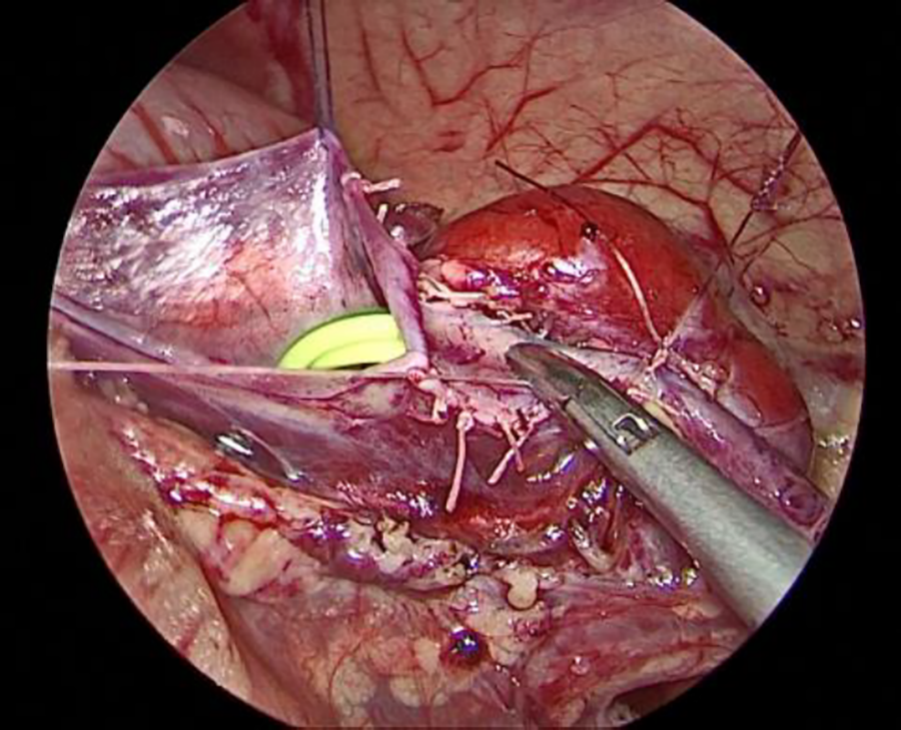
Suturing the posterior wall of the ureter.

The child was placed in a supine position, and the affected side was elevated at 30 degrees. A small incision of approximately 0.5 cm was created in the skin around the umbilicus. Then, a 5-mm abdominal puncture instrument was employed under direct visualization to create pneumoperitoneum, and the intra-abdominal pressure was adjusted to 9 mmHg. Further, two additional 3-mm trocars were placed via laparoscopic guidance at the midline of the abdominal wall, approximately 3 cm above and below the umbilicus (Figure 1). The laparoscope was introduced into the abdominal cavity to visualize the renal pelvis as an oval-shaped dilation behind the peritoneum. Using an electric hook, the mesentery and peritoneum were incised by approximately 2 cm to expose the dilated renal pelvis and ureter. The ureteropelvic junction displayed constricted and tortuous. Of note, 3-mm straight scissors were employed to transect the narrow segment of the distal ureter, and a 1-cm vertical incision was created on the outer wall of the distal ureter. Then, a 4.0 absorbable suture was employed to lift the renal pelvis 1 cm from the lower pole of the kidney (Figure 2), and the excess renal pelvis tissue was excised using a 3-mm straight scissor. A 5–0 absorbable suture was subsequently used to intermittently and obliquely anastomose the lowest point of the V-shaped renal pelvis at the lower pole of the kidney to the lowest point of the distal ureter where it was incised (Figure 3). The width of the anastomosis was approximately 1.5:1 between the renal pelvis and the ureter to maintain tension and create a funnel-shaped anastomosis (Figure 4). A 4.7 *F* × 14 cm ureteral stent was placed in the anterior wall of the ureter (Figure 5). Then, the posterior wall of the ureter was sutured (Figure 6). The remaining renal pelvis was sutured continuously using 4–0 absorbable sutures. The renal pelvis and ureter were repositioned after obtaining adequate hemostasis behind the peritoneal cavity, and the incision was closed layer by layer.

Postoperatively, the child was kept on nothing by mouth (NPO) for 12–24 h, followed by the resumption of breastfeeding. Routine fluid replacement and vitamin K supplementation were administered. Antibiotics were used for 5–7 days. The abdominal drainage tube was removed three days post-surgery, and a double J stent was left in place for 1–3 months.

## 4 Statistical analysis

Statistical analysis was conducted using SPSS 18.0. Metric data, including anteroposterior diameter of the renal pelvis (cm) and renal parenchymal thickness (cm), were presented as mean ± standard deviation (*χ* ± s). Statistical analysis was performed using repeated measures analysis of variance.

## 5 Results

We included 16 pediatric patients with severe PNH confirmed by ultrasonography screening, which included 13 male and 3 female patients. In total, 13 cases were present on the left side and 3 on the right side. The average age at the time of surgery was 9.50 days (ranging from 8.50–12.00 days), and the average weight was 3.30 ± 0.95 kg. All patients underwent laparoscopic pyeloplasty without the requirement for conversion to open surgery. The average duration of surgery was 292.06 ± 73.60 min, with an average blood loss of 2.50 ml (ranging from 2.00–5.00 ml). The postoperative mean length of hospital stay was 13.44 ± 4.70 days (Table 1). No cases of anastomotic leakage were reported, and follow-up visits were conducted at 1 month, 3 months, 6 months, and 12 months postoperatively, respectively. No cases of double J stent displacement were observed. One patient had the stent removed 1 month after surgery, and the remaining patients had the stent removed 3 months after surgery. Postoperative examinations, including urinalysis and US, did not show any worsening of hydronephrosis. One patient exhibited renal atrophy upon reexamination six months post-surgery. Changes in renal pelvis anteroposterior diameter exhibited a time effect (*F* = 49.281, *P *< 0.001), with significant differences at 1, 3, 6, and 12 months postoperatively compared to preoperative values (*P *< 0.05) (Table 2). Significant differences were also noted between 6 and 1 month, as well as between 12 and 1 month postoperatively (*P *< 0.05) (Table 3).

**Table 1:** Basic information of surgery.

Variable	Value
Surgical age (days)	9.50 (8.50–12.00)
Weight (kg)	3.30 ± 0.95
Surgery duration (min)	292.06 ± 73.60
Blood loss (ml)	2.50 (2.00–5.00)
Postoperative hospital stay (days)	13.44 ± 4.70

**Table 2:** Repeated measures analysis of variance for anteroposterior diameter of the renal pelvis.

	Renal pelvis anteroposterior diameter (cm)	*F*	*P*
Preoperative	3.52 ± 0.69	49.281	<0.001
1 month postoperative	1.22 ± 0.60^a^		
3 months postoperative	1.54 ± 0.99^a^		
6 months postoperative	1.11 ± 0.53^a,b^		
12 months postoperative	0.97 ± 0.33^a,b^		

^a^ Compared to preoperative values, *P *< 0.05.

^b^ Compared to 3 months postoperative, *P *< 0.05.

**Table 3:** Repeated measures analysis of variance for renal parenchymal thickness.

	Anteroposterior diameter of the renal pelvis (cm)	*F*	*P*
Preoperative	0.22 ± 0.07	9.516	<0.001
1 month postoperative	0.33 ± 0.11^a^		
3 months postoperative	0.35 ± 0.17^a^		
6 months postoperative	0.39 ± 0.10^a,b^		
12 months postoperative	0.43 ± 0.17^a,b^		

^a^ Compared to preoperative values, *P *< 0.05.

^b^ Compared to 1 month postoperative, *P *< 0.05.

According to repeated measures analysis of variance, there is a time effect in the changes of the anteroposterior diameter of the renal pelvis (*F* = 49.281, *P *< 0.001). Post hoc tests revealed statistically significant differences at 1 month, 3 months, 6 months, and 12 months postoperatively compared to preoperative values (*P *< 0.05). Additionally, there were statistically significant differences between 6 months and 3 months postoperatively, as well as between 12 months and 3 months postoperatively (*P *< 0.05).

According to repeated measures analysis of variance, there is a time effect in the changes of renal parenchymal thickness (*F* = 49.281, *P *< 0.001). Post hoc tests revealed statistically significant differences at 1 month, 3 months, 6 months, and 12 months postoperatively compared to preoperative values (*P *< 0.05). Additionally, there were statistically significant differences between 6 months and 1 month postoperatively, as well as between 12 months and 1 month postoperatively (*P *< 0.05).

## 6 Discussion

**Figure 7:**
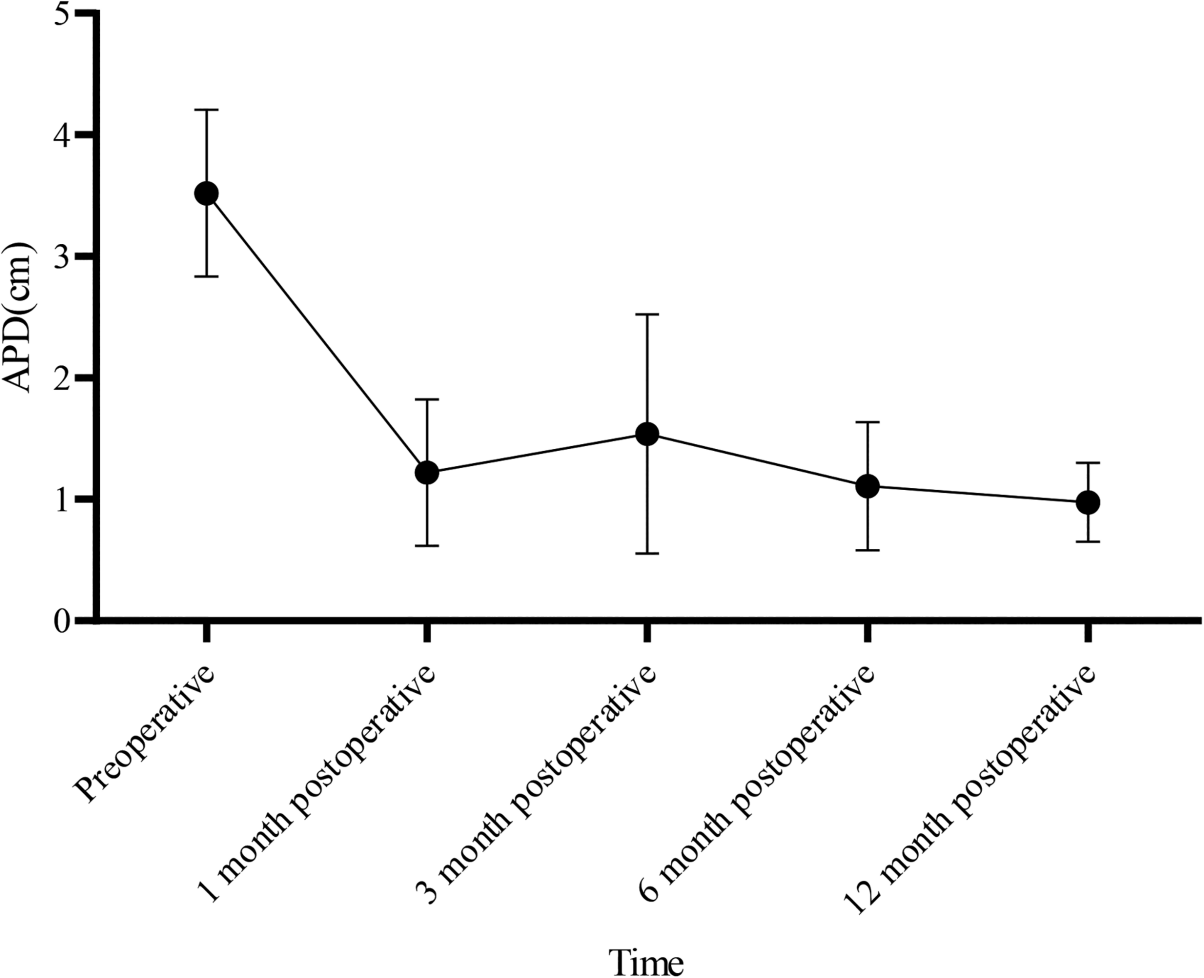
The changes in the anteroposterior diameter of the renal pelvis over time.

**Figure 8:**
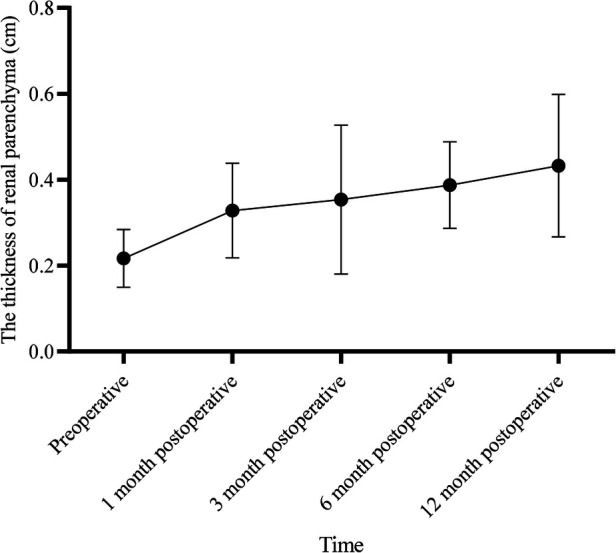
Changes in renal parenchymal thickness over time.

PNH is the most prevalent cause of renal and urinary tract abnormalities during the perinatal period with an incidence rate of 1%–5% (1, 2). UPJO is a leading cause of neonatal hydronephrosis (5), and approximately 85%–90% of PNH cases occur due to organic lesions. Many researchers believe that UPJO in newborns can be managed conservatively. However, the timing of surgical intervention is still debatable. The most aggressive viewpoint suggests performing prenatal surgery to address the problems of the resolution of the urinary system and prevent renal damage. However, prenatal US cannot distinguish between obstructive and non-obstructive causes of hydronephrosis. Therefore, prenatal surgery is less practical and can be potentially harmful to the fetus and the mother (6). Nonetheless, in cases of severe hydronephrosis or ongoing deterioration of renal function, surgery is required. Several previous studies have determined the outcomes of children with PNH who undergo conservative treatment (CT) or early surgical treatment (EST) after birth. Early obstruction is characterized by inflammation cell infiltration and dilatation of renal tubules, which can gradually cause renal dysfunction (7). Arora et al. (8) reported that EST promotes the recovery of renal function in severe hydronephrosis patients, whereas delayed surgery can only partially restore the lost renal function. Therefore, surgery is the preferred method for treating severe hydronephrosis. Studies by Deng et al. (9) reported that during the diagnosis and treatment of severe hydronephrosis, EST can promote the recovery of renal structure and function, and CT may cause worsening of renal functions. In 2014, Policiano et al. (10) reported that an APD of over 10 mm in the prenatal US is an indication of surgical intervention. Vemulakonda et al. (11) reported that any fetus with an APD of 10 mm or more qualifies as having severe PNH, regardless of gestational age, and should be monitored closely. Safai Asl and Maleknejad proposed that an APD of 15 mm or more is the optimal cutoff point for surgery with a sensitivity of 88% and specificity of 74% (12, 13). Yiee et al. (14) outlined the criteria for fetal hydronephrosis surgery, which were as follows: (1) APD > 3 cm; (2) APD > 2 cm with concurrent calyceal dilation; (3) split renal function <30%; (4) worsening of renal functions; (5) aggravation of hydronephrosis; and (6) symptomatic hydronephrosis. These criteria were accepted by the American Academy of Pediatrics (AAP) in 2010 as markers for early surgical intervention (15). All cases in this study presented severe hydronephrosis in the neonatal period, with an APD > 2 cm (3.52 cm ± 0.69 cm) and SFU grade 4, which is consistent with the surgical criteria. There was no instance of recurrence among the 16 cases in this study. For severe PNH, early surgical intervention not only relieved the renal hydronephrosis significantly compared with preoperative conditions but also increased renal parenchymal thickness. Moreover, there were statistically significant differences between 6 months and 3 months postoperatively, as well as between 12 months and 3 months postoperatively (*P *< 0.05). This indicates that with longer postoperative follow-up, there is a better recovery of hydronephrosis (Figure 7), and the renal parenchymal thickness increases (Figure 8). However, in one case, renal atrophy was found during a follow-up examination six months post-surgery. This patient had an APD of 2.5 cm and a renal cortex thickness of 0.14 cm before surgery, with a cystic appearance of the kidney, indicating severe hydronephrosis. Even after relieving the obstruction in the neonatal period, renal atrophy can present as a risk. Therefore, early surgery should be considered for severe neonatal hydronephrosis to mitigate damage to renal function, especially in cases where the renal cortex is significantly thin.

Anderson-Hynes (A-H) dismembered pyeloplasty was first introduced by Anderson and Hynes in 1949 (16). Since then, A-H dismembered pyeloplasty has been recognized as the preferred surgical procedure for treating UPJO. Open surgery for UPJO is associated with characteristics, such as large incisions, noticeable scarring, suboptimal cosmetic results, and slow postoperative recovery. With the advancements and developments in laparoscopic surgical techniques, Peters reported the first case of laparoscopic A-H pyeloplasty in children in 1995. Laparoscopic A-H pyeloplasty has increasingly been used in clinical practice due to its advantages such as smaller incisions, shorter hospital stays, improved cosmetic outcomes, and good clinical results (17), which has now become the preferred surgical method for treating UPJO. However, laparoscopic pyeloplasty has technical limitations, especially in newborns, because the operative space is limited, surgical times can be longer, and there is a possibility of using minimally invasive approaches for pyeloplasty in newborns. Therefore, laparoscopic pyeloplasty in infants is more technically challenging, and laparoscopic techniques are only employed in a few healthcare institutions (18, 19). Reed et al. (20) reported successful outcomes in 123 infants under the age of one who underwent laparoscopic pyeloplasty at nine different centers. The average weight of the operated infants was 6.8 kg, and the smallest weight was 3 kg. Among these cases, five infants experienced anastomotic fistulas. In this study, the average weight of the infants was 3.3 kg, and the smallest weight was 2.5 kg. All surgeries were performed laparoscopically, and no anastomotic fistula-associated complication was observed. This indicates that surgery can be performed in infants with lower body weight as well. Long-term postoperative US follow-up was conducted, and double J stents were left for three months. The patients were administered standard prophylactic oral cefuroxime to prevent infections, and urinary leukocyte levels were monitored. One patient experienced a febrile urinary tract infection one month postoperatively, and the ureteral stent was removed after administering antibiotics. The surgery was performed through very small incisions, with the incision for the camera lens located at the navel, leading to excellent cosmetic outcomes. Infant ureters are narrower, making laparoscopic pyeloplasty more challenging and time-consuming. The entire surgical procedure, including the anastomosis of the renal pelvis and ureter and the placement of the double J stent, was performed laparoscopically. Maintaining a 1.5:1 width ratio between the renal pelvis and the ureter during intermittent suturing allows the ureteral valve to maintain tension, resulting in a wider and smoother anastomosis. This approach can also address the issue of continuous suturing leading to tissue contraction and helps avoid larger anastomotic fistulas. Inserting the double J stent is an important part of the surgery. After completing the anterior wall anastomosis, a 4.7 F double J stent was inserted under the guidance of a guide wire and aspirator (Figure 5). It was not necessary to pull the anastomosis out of the abdominal cavity to avoid affecting it. After the guide wire entered the bladder, care was taken to pull the guide wire, allowing the balloon to about the urethral bladder outlet to prevent the guide wire from extending out of the urethra, which can affect the placement of the double J stent.

Sun et al. (21) reported that robotic-assisted laparoscopic pyeloplasty has the advantages of less trauma and faster recovery than laparoscopic pyeloplasty in 33 patients (age: 0–36 months) with UPJO. It can be safely and effectively performed in infants and young children, and its effectiveness is similar to that of traditional conventional laparoscopic pyeloplast. Li et al. (22) also retrospectively analyzed 9 infants under 3 months who underwent robotic-assisted laparoscopic pyeloplasty for severe UPJO and showed an overall complication rate of 22%, a median operational time (OT) of 109.5 (±10.4) min, and a length of hospitalization of 5.57 (±0.73) days in their series. In our present work, the average duration of surgery was 292.06 ± 73.60 min, and the postoperative mean length of hospital stay was 13.44 ± 4.70 days. These outcomes seem to be high as compared with robotic-assisted laparoscopic pyeloplasty. This difference may be related to the younger age of the patients in this study. However, Due to the high cost of robots, robotic-assisted laparoscopic pyeloplasty have not been widely used in all children's hospitals, and the medical expenses are relatively high. At the same time, it increases the burden on the families of patients. In order to achieve the same effect and reduce the burden on the families of patients, even if the surgery time is too long, it is still a good choice.

## 7 Conclusion

Laparoscopic pyeloplasty is a safe and effective approach with minimal complications for obstructive severe neonatal hydronephrosis at the ureteropelvic junction. As the postoperative follow-up duration increases, there is a better recovery of hydronephrosis, and the renal parenchymal thickness increases. However, this study has some limitations. This is a retrospective study with a small sample size, which was performed at a single center. More extensive research is warranted to further assess the efficacy and complications of this procedure. Furthermore, the follow-up period in this study was relatively short, and longer-term follow-up is required to evaluate the long-term outcomes.

## Data Availability

The raw data supporting the conclusions of this article will be made available by the authors, without undue reservation.
